# Dormancy and germination of the trimorphic achenes of a cold desert annual: spreading the risk over time

**DOI:** 10.1093/aobpla/plaa056

**Published:** 2020-10-23

**Authors:** Juanjuan Lu, Wenjing Dong, Dunyan Tan, Carol C Baskin, Jerry M Baskin

**Affiliations:** 1 College of Grassland and Environment Sciences, Xinjiang Agricultural University, Urümqi, China; 2 Department of Biology, University of Kentucky, Lexington, KY, USA; 3 Department of Plant and Soil Sciences, University of Kentucky, Lexington, KY, USA

**Keywords:** Asteraceae, diaspore heteromorphism, germination phenology, high risk/low risk dispersal–dormancy strategy, physiological dormancy, temporal dispersal, unpredictable-precipitation environment

## Abstract

Many studies have been done on the relationship between variation in morphology, dispersal ability and degree of dormancy of heterocarpic species with dimorphic diaspores. However, there are far fewer such studies on species that produce trimorphic diaspores. Our aim was to compare dormancy and germination of achenes from peripheral, intermediate and central positions in the capitulum of the diaspore-trimorphic cold desert annual Asteraceae species *Heteracia szovitsii*, an important component of plant communities in the cold deserts of NW China. Dormancy breaking/germination responses of the three achene morphs and of seeds isolated from the pericarp were tested in the laboratory using standard procedures, and seedling emergence phenology of the achene morphs was monitored under natural cold desert temperature conditions in an experimental garden with and without supplemental watering. Depth of dormancy of the three achene morphs was peripheral > intermediate > central. Seedlings from the three morphs emerged in spring and in autumn. Cumulative seedling emergence percentage from achenes during 47 months of burial was central > intermediate > peripheral. Central achene morphs emerged over a period of ~12 months after sowing, while intermediate and peripheral achene morphs did so for ~40 and 47 months, respectively. Thus, *H. szovitsii* exhibits a temporal dispersal strategy. No viable central or intermediate achene morphs were present after 16 and 40 months, respectively, but ~60 % of the non-emerged peripheral achenes morphs were viable after 47 months. Based on our results on diaspore dormancy and those of a previous study of diaspore spatial dispersal of *H. szovitsii*, we conclude that this species has a high–intermediate–low risk diaspore dispersal/dormancy strategy that likely increases the chances for population persistence over time and space.

## Teaser

Relatively little is known about the relationship between variation in morphology, degree of dormancy and dispersal ability of heterocarpic species with trimorphic diaspores. Lu *et al.* investigated diaspore dormancy of *Heteracia szovitsii* and combined the results with data from a previous study of diaspore dispersal of this species. They concluded that *H. szovitsii* has a high–intermediate–low risk diaspore dispersal/dormancy strategy, which likely increases the chances for population persistence over time and space in its cold desert habitat in Central Asia.

## Introduction

Diaspore heteromorphism is a phenomenon in which fruits/seeds that differ in morphology and/or ecological responses such as dormancy and dispersal are produced by an individual plant. Variation in dormancy and dispersal spreads the risk of plant establishment over time and space, respectively. It is an adaptative strategy that maximizes the probability for persistence of a species in a spatio-temporally variable habitat (Venable and Lawlor 1980; Venable 1985; Mandák 1997; Imbert 2002; Baskin *et al.* 2014). Diaspore heteromorphism has been described in ~300 species in 26 families of phylogenetically advanced angiosperms, and it is common in Amaranthaceae, Asteraceae, Brassicaceae and Poaceae (Wang *et al.* 2010; Scholl *et al.* 2020). The diaspores may differ in size, shape, colour, mass and accessory structures, as well as in ecological attributes such as dispersal, dormancy/germination characteristics and type of seed bank (i.e. persistent vs. transient). Further, plants that arise from heteromorphic diaspores may differ ecologically with respect to survival/establishment, growth, reproduction and tolerance to abiotic and biotic factors of the environment. Many studies have been conducted on the ecology of species with dimorphic diaspores (reviewed in Baskin *et al.* 2013, 2014). However, there are fewer such studies on species that produce three diaspores (Venable *et al.* 1987; Wei *et al.* 2007; Wang and Wei 2007; Cheng and Tan 2009; Sun *et al.* 2008, 2009; Ma *et al.* 2018; Mamut *et al.* 2018; Wang *et al.* 2019, 2020).

*Heteracia szovitsii* (Asteraceae) is an annual ephemeral species that occurs in Central Asia, Iran, Turkey and Caucasia (An *et al.* 1999). It is the only species in the genus *Heteracia*, and in China the species occurs only in the southern part of the Junggar Basin, Xinjiang. This species is one of the common ephemeral species that germinates in early spring in NW China (Mao and Zhang 1994; Wang *et al.* 2006). It is an important component in plant communities of the cold deserts in this area (including sandy deserts, gravel deserts and desert grassland) and is ecologically significant in maintaining plant diversity and stability of the cold desert ecosystem (Mao and Zhang 1994; An *et al.* 1999).

**Figure 1.**
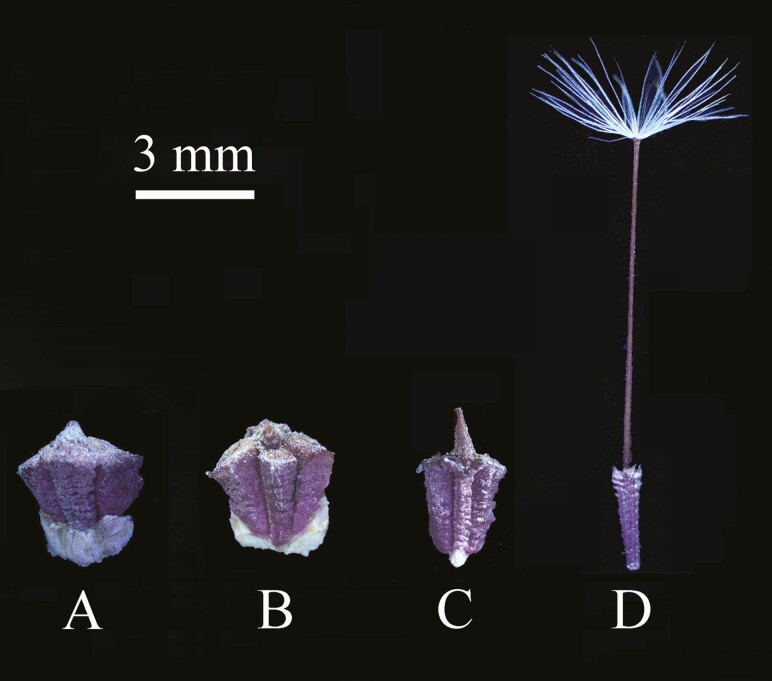
Morphological characteristics of the three achene morphs of *Heteracia szovitsii*. (A, B) Peripheral achene morph (PA; A, abaxial side; B, adaxial side); (C) intermediate achene morph (IA); (D) central achene morph (CA).

During our field observations over several years, we have found only a few seedlings in autumn but many in spring (March and April). Plants begin to flower in late April or early May, and most of them senesce by early to mid-June. Plants produce three achene morphs in each inflorescence (capitulum), i.e. peripheral (Fig. 1A and B), intermediate (Fig. 1C) and central (Fig. 1D). The peripheral achene morphs are wide with no pappus (Fig. 1A and B), intermediate achene morphs short with no pappus (Fig. 1C) and central achene morphs elongated with pappus (Fig. 1D) (Cheng and Tan 2009). Dispersal and germination units of both the central and intermediate morphs are achenes, while those of the peripheral morph are the achene with an attached bracteole (Cheng and Tan 2009).

According to the Nikolaeva–Baskin classification system, there are five classes of seed dormancy (Baskin and Baskin 2004, 2014). Physiological dormancy (PD) is caused by low growth potential of the embryo, physical dormancy (PY) by a water-impermeable seed or fruit coat, combinational dormancy (PY + PD) by a water-impermeable seed (or fruit) coat and low growth potential of the embryo, morphological dormancy (MD) by an underdeveloped embryo that needs to complete growth (the dormancy period) within the mature seed before the radicle emerges (i.e. seed germinates) and morphophysiological dormancy (MPD) by an underdeveloped embryo that also is physiologically dormant. Of these, the only class of dormancy known to occur in seeds of Asteraceae is PD. Physiological dormancy occurs in three increasing degrees or depths (intensities) of dormancy: non-deep PD < intermediate PD < deep PD.

Dry storage (after-ripening) and warm plus cold stratification broke dormancy of central achene morphs (Cheng 2009). Dormancy of intermediate achene morphs was broken by pericarp removal and by pericarp removal plus treatment with gibberellic acid (GA_3_), while dormancy of peripheral achene morphs was broken only by pericarp removal plus treatment with GA_3_. Thus, the central achenes of *H. szovitsii* clearly have non-deep PD. However, the intermediate and peripheral achenes have an unknown level of PD that is more difficult to break than the non-deep PD of the central achenes (Cheng 2009). Baskin *et al.* (2014) suggested that the intermediate and peripheral achene morphs of *H. szovitsii* might have intermediate PD but noted that the peripheral achenes are more dormant than the intermediate ones.

We hypothesized that (i) the intermediate and peripheral achene morphs of *H. szovitsii* have intermediate and/or deep PD, and (ii) the three achene morphs produced by a cohort of plants in a given year spread germination over a period of several years, resulting in multiple germination and seedling establishment events, i.e. dispersal in time. To test these hypotheses, we (i) compared dormancy breaking and germination requirements of the three achene morphs in the laboratory, and (ii) monitored their emergence under natural cold desert temperature conditions with and without supplemental watering.

This supplemental watering comparison was included because if the soil is sufficiently moist to promote germination of annuals in autumn in the cold desert, seeds germinate and surviving plants have a winter annual life cycle. If the soil is too dry for seeds to germinate in autumn, germination is delayed until spring, at which time the soil is moist due to rain and/or meltwater from snow (Wang *et al.* 2006), and seeds germinate and plants behave as ephemerals (Lu *et al.* 2014). In years with a reasonable amount of rainfall in autumn, a portion of the seed population of an annual species germinates in autumn and another portion in spring (Lu *et al.* 2014, 2017), i.e. the species behaves as a facultative winter annual. Thus, our water-addition treatments were used to compare autumns with and without a reasonable amount of precipitation.

## Materials and Methods

### Field site description and achene collection

Freshly matured achenes were collected from several hundred plants of *H. szovitsii* growing in a natural population on Yamalic Hill, a western suburb of Urümqi city on the southern edge of the Junggar Basin of Xinjiang, China (43°79′N, 87°56′E, 900 m a.s.l.). Achenes from the capitula were bulked into one collection, separated into three morphs and stored at ambient conditions in the laboratory in paper bags until used. Photographs of the three achene morphs (Fig. 1) were taken using a camera mounted on a Nikon SMZ1000 Digital Stereoscopic Microscope. Unless otherwise stated, the following experiments were conducted using achenes collected on 17 June 2016.

The southern edge of the Junggar Basin has cold desert vegetation dominated by Amaranthaceae (subfam. Chenopodioideae) and Asteraceae, gravelly grey desert soil and a continental climate. For the years 1991–2019 at Urumqi, mean annual temperature was 7.7 °C, and extreme mean monthly temperature of the coldest (January) and hottest (July) months were −15.7 °C and 28.9 °C, respectively. Average annual precipitation was 264.2 mm and ranged from 48.3 to 419.5 mm, with a coefficient of variation (CV) of 38.7 % (National Meteorological Information Center, China Meteorological Administration). In particular, then, the amount of annual precipitation is highly variable among seasons and years, but generally rainfall is higher in spring than in autumn (Wang *et al.* 2006). Further, water from snowmelt increases water availability in spring. The annual potential evaporation is >2000 mm (Wei *et al.* 2003).

### Experimental design

#### Part I: Imbibition by achenes and by seeds within them.

Water uptake (imbibition) was determined for the three achene morphs that had been stored dry at laboratory conditions for 20 days. Four replicates of 25 achenes of each morph were placed on Whatman No. 1 filter paper moistened with distilled water in 9-cm-diameter Petri dishes and kept on a laboratory bench at room conditions (ca. 28 °C, 46–50 % RH). At time 0 and every 10 min until they reached final constant mass, achenes were removed from the dishes, blotted dry with filter paper, weighed to the nearest 0.0001 g with an electronic balance and returned to the Petri dishes.

When water uptake by the three achenes morphs reached a plateau, percentage increase in mass (% *W*_r_) for each morph was calculated as % = [(*W*_i_ − *W*_d_)/*W*_d_] × 100, where *W*_i_ and *W*_d_ = mass of imbibed and air-dry morphs, respectively. Intact imbibed and non-imbibed (control) achenes of the three morphs were cut open, and seeds were removed from them and weighed. Mass of seeds dissected from imbibed and non-imbibed achenes was compared.

#### Part II: Germination and dormancy.

The three achene morphs were tested for germination in light (12 h of ≈100 µmol m^−2^ s^−1^, 400–700 nm, cool white fluorescent light each day) and in constant darkness (Petri dishes with achenes in them placed in light-proof black bags) at 12/12 h daily temperature regimes of 5/2, 15/2, 20/10 and 30/15 °C. The 5/2 °C regime represents early and late winter, 15/2 °C early spring and late autumn, 20/10 °C late spring and early autumn and 30/15 °C summer (Sun *et al.* 2009). Achenes (central and intermediate) or achenes plus bracteole (peripheral) (hereafter, central, intermediate and peripheral achene morphs) were incubated in 9-cm-diameter Petri dishes on two layers of Whatman No. 1 filter paper moistened with distilled water, which was added as needed to keep the filter paper moist. Unless otherwise stated, four replications of 25 achenes or 25 seeds (pericarp removed from achenes) each were used for all experiments. The criterion for germination was emergence of the radicle from the achene (or seed). Germination in light was monitored daily for 28 days, and germinated achenes/seeds were removed at each counting. Achenes (or seeds) incubated in dark were checked only after 28 days. After the germination trials were completed, non-germinated achenes/seeds were checked for viability. Achenes/seeds with white, firm embryos were counted as viable and those with soft grey embryos as non-viable.

##### Germination of fresh achenes and their excised seeds.

###### Germination of fresh achenes.

To determine if fresh intact achenes are dormant, the three achenes morphs were incubated at the four temperature regimes in light and in darkness for 28 days, beginning on 20 June 2016.

###### Germination of seeds excised from fresh achenes.

To determine if dormancy of fresh achenes is due to presence of the seed covering layers, the pericarp and bracteole were removed from the peripheral achene morph and the pericarp was removed from intermediate and central achene morphs. Seeds were excised from covering layers on 23 June 2016 and incubated at the four temperature regimes in light and in darkness for 28 days, as described above.

##### Effect of light/dark and temperature on dormancy break of achenes.

To determine the effect of prolonged incubation over the range of temperature regimes in light and in darkness on dormancy break and germination, the three achene morphs were placed at the four temperature regimes in light and in darkness. All dishes were checked for germinated achenes at 1-week intervals for 12 months (those in dark using a green safe light), at which time any seedlings present were removed and watered added to dishes if needed. The green light source consisted of a 5-W cool white incandescent tube wrapped with an acrylic green sheet (Shandong Luke Electric Appliance Co., Ltd, Shandong, China). The peak intensity of green light as determined using a PR-650 spectrascan colorimeter (Photo Research, Inc., Chatsworth, CA, USA) was at 530 nm. The average photon flux density of green light at seed level, determined with a LI-6400 portable photosynthesis system (LI-COR Company, Lincoln, NE, USA) was ~3 μmol m^−2^ s^−1^.

##### Effect of dry storage (after-ripening) on dormancy break of achenes.

The purpose of this study was to compare dormancy break of the three achene morphs that has been dry-stored at room conditions for 0, 6, 12, 18, 24, 30, 36, 42 and 48 months, after which they were tested for germination at the four temperature regimes in light for 28 days.

##### Effect of cold and/or warm stratification on dormancy break of achenes.

The purpose of this experiment was to determine if cold and/or warm stratification was (were) required to break dormancy of the three achene morphs.

###### Cold stratification.

Achenes of the three morphs that had been stored dry at room conditions for 0, 12 and 24 months were cold-stratified on moist filter paper at 4 °C in constant dark for 0, 4, 8, 12, 16 and 20 weeks. After each cold stratification period, achenes were incubated at the four temperature regimes in light for 28 days. No achenes germinated during cold stratification.

###### Warm stratification.

Achenes of the three morphs that had been stored dry at room conditions for 0, 12 and 24 months were warm-stratified on moist filter paper at 30/15 °C in constant dark for 0, 4, 8, 12, 16 and 20 weeks. After each warm stratification period, achenes were incubated at the four temperature regimes in light for 28 days. No achenes germinated during warm stratification.

###### Warm plus cold stratification.

Freshly collected achenes of the three morphs were kept in constant darkness at 30/15 °C on both wet and dry filter paper for 4 weeks. Then, they were kept on moist filter paper in constant darkness at 4 °C for 4, 8 and 12 weeks. After each of the three cold stratification periods, achenes were incubated at the four temperature regimes in light for 28 days. No achenes germinated during the treatments.

##### Effect of pericarp scarification on dormancy break of achenes.

To determine the effect of pericarp scarification on dormancy break, the three achene morphs stored dry at room conditions for 0, 6 and 12 months were scarified by removing a small piece of the pericarp adjacent to the cotyledon end of the seed with a razor blade. Then, scarified and non-scarified (intact) achenes were incubated at the four temperature regimes in light for 28 days.

##### Effects of GA_3_ and pericarp scarification on dormancy break of achenes.

To determine the effects of GA_3_ on dormancy break, intact and scarified achenes of the three morphs that had been stored dry at room conditions for 0, 6 and 12 months were placed on filter paper moistened with 0 (distilled water control) or with 0.1, 1.0 and 10.0 mmol L^−1^ GA_3_ solutions and incubated at the four temperature regimes in light for 28 days. Gibberellic acid was provided by Shanghai Yuanye Bio-Technology Co., Ltd, Shanghai, China.

##### Effect of temperature sequence on dormancy break of achenes.

To determine if warm plus cold or cold plus warm stratification is required for dormancy break of the three achene morphs, a move-along experiment (Baskin and Baskin 2003) was conducted. For each morph, 50 achenes were sown on wet filter paper in each of 24 Petri dishes. Four dishes of each morph were incubated continuously at each of the four temperature regimes in light to serve as controls. In the move-along portions of the experiment (treatments), four dishes of 50 achenes of each morph was placed in light at the following two sets of temperature regimes: (i) cold plus warm [i.e. 5/2 °C (12 weeks) → 15/2 °C (4 weeks) → 20/10 °C (4 weeks) → 30/15 °C (12 weeks) → 20/10 °C (4 weeks)], and (ii) warm plus cold [i.e. 30/15 °C (12 weeks) → 20/10 °C (4 weeks) → 15/2 °C (4 weeks) → 5/2 °C (12 weeks) → 15/2 °C (4 weeks)]. The total number of dishes was 72 [i.e. 4 dishes/replications × 3 achene morphs × 6 temperature treatments (i.e. 4 continuous temperatures + 2 move-along temperatures)].

##### Effect of scarification and dry storage on dormancy break of peripheral and intermediate achene morphs.

Germination percentages were determined in June 2018 for scarified peripheral and intermediate achene morphs stored dry at laboratory conditions for 0 months (collected in 2018), 12 months (2017), 24 months (2016), 36 months (2015) and 48 months (2014). We did not determine if there was a maternal effect on seeds collected in the 5 years, but we have no reason to suspect that there was a year-of-collection effect. Achenes were scarified by removing a small piece of the pericarp on the cotyledon end with a razor blade. Non-scarified (intact) and scarified achenes and excised seeds (pericarps removed) of the two morphs were placed on Whatman No. 1 filter paper moistened with distilled water in 9-cm-diameter Petri dishes and incubated at 5/2 °C in light for 28 days.

##### Effect of cold stratification and scarification on dormancy break of peripheral and intermediate achene morphs.

Fresh (collected in June 2018) peripheral and intermediate achene morphs were subjected to three treatments: (i) intact achenes (not scarified) cold-stratified (control); (ii) pericarp scarified and then achenes cold-stratified; and (iii) pericarp scarified after intact achenes were cold-stratified for each cold stratification period. In this experiment, achenes were cold-stratified on moist filter paper at 4 °C in constant darkness for 0, 4, 8, 12, 16 and 20 weeks.

After each period of cold stratification, four replications of 25 achenes from both morphs were checked for germination. Then, four replicates of 25 achenes of both morphs that did not germinate during cold stratification were incubated at 5/2 °C in light for 28 days.

#### Part III: Emergence phenology of the three achene morphs in an experimental garden.

The purpose of this experiment was to compare the timing of emergence of the three achene morphs under natural temperature conditions with and without supplemental watering. This study was conducted in an experimental garden on the campus of Xinjiang Agricultural University in Urumqi, near the southern edge of the Junggar Basin. On 25 June 2016, 50 achenes of each of the three morphs of *H. szovitsii* collected on 17 June 2016 were sown at a depth of 0.5 cm in 10 clay pots (20 cm deep and 24 cm in diameter) filled with a mixture of 70 % grey desert soil and 30 % desert sand; sand was added to improve soil aeration and drainage. All pots were placed on the soil surface in the experimental garden. For each of the three achene morphs, five pots were watered and the other five not watered [5 replications × 3 achene morphs × 2 watering treatments (i.e. watered and non-watered) = 30 pots]. In the watering treatment, the soil was watered to the field capacity every 3 days throughout the experiment, except during winter (December to February), when the soil was frozen. The non-watered soil received water only via rainfall and snowmelt. Seedling emergence was monitored at 7-day intervals from sowing to 10 May 2020.

To monitor viability of the three achene morphs, three replications of 10 achenes for each of the three morphs (3 replications × 10 achenes × 3 achene morphs) were placed in mesh bags and buried at a depth of 0.5 cm in soil in the experimental garden. Bags were retrieved directly from soil at the end of experiments in each germination season (autumn and spring 2016–19), and seed viability was tested as described above. Information on temperature and rainfall at the study site (Urumqi) was obtained from data collected at the National Meteorological Information Center, China Meteorological Administration **[see****Supporting Information—Fig. S1****]**.

### Statistical analyses

Independence of the replications was analysed for germination data by chi-square test, and the full independence of all replications are shown. Then, all data were analysed for normality and homogeneity of variance prior to analysis to fulfil requirements of *t*-tests and ANOVAs. If data were normal and homogeneous, they were subjected to further analysis. If data were not normally distributed or if variances were not homogeneous, they were arcsine (square root %) transformed (germination percentage data) or log_10_ transformed (dry and imbibed mass) before further analysis to ensure homogeneity of variance. Non-transformed data appear in all figures.

Paired-sample *t*-tests were used to compare mass of each of the three achene morphs and of seeds within them before and after imbibition. When variances of non-transformed or transformed data were homogeneous, a one-way ANOVA was used to determine differences in germination percentage of the three achene morphs and of seeds among the treatments. Tukey’s HSD test was performed for multiple comparisons to determine significant differences among the treatments.

Two-way ANOVAs were used to test for significance of main effects [pericarp treatment (i.e. intact and scarified), storage time] and their interactions on germination of peripheral and intermediate achene morphs in the ‘*Effect of scarification and dry storage on dormancy break of peripheral and intermediate achenes*’ experiment and for significance of main effects (cold stratification time and pericarp treatment) and their interactions on germination peripheral and intermediate achene morphs in the ‘*Effect of cold stratification and scarification on dormancy break of peripheral and intermediate achenes*’ experiment. Three-way ANOVAs were used to test for significance of main effects (achene morph, light and temperature) and their interactions on germination in the ‘*Germination of fresh achenes and their seeds*’ experiment, for significance of main effects (achene morph, storage time and temperature) and their interactions on germination in the ‘*Effect of dry storage (after-ripening) on dormancy break of achenes*’ experiment and for significance of main effects [achene morph, watering treatment (i.e. watered and non-watered) and germination season] and their interactions on germination in the ‘*Emergence phenology of the three achene morphs in an experimental garden*’ experiment. Four-way ANOVAs were used to test for significance of main effects (achene morph, temperature, light and incubation time) and their interactions on germination in the ‘*Effect of light/dark and temperature on dormancy break of achenes*’ experiment and for significance of main effects (achene morph, temperature, storage time and pericarp treatment) and their interactions on germination in the ‘*Effect of pericarp scarification on dormancy break of achenes*’ experiment. A five-way ANOVA was used to test for significance of main effects (achene morph, temperature, dry storage time, stratification time and stratification treatment) and their interactions on germination in the ‘*Effect of cold and/or warm stratification on dormancy break of achenes*’ experiment and for significance of main effects (achene morph, temperature, storage time, pericarp treatment and GA_3_ concentration) on germination in the ‘*Effect of GA*_*3*_*and pericarp scarification on dormancy break of achenes*’ experiment. Statistical tests were conducted at *P* = 0.05. All data analyses were performed with the software SPSS 19.0 (SPSS Inc., Chicago, IL, USA).

## Results

### Part I: Imbibition by achenes and by seeds within them

Before imbibition, the dry mass of 25 peripheral, intermediate and central achene morphs was 139.5 ± 4.9 mg, 39.7 ± 0.2 mg and 22.2 ± 0.6 mg, respectively. The dry mass of 25 seeds excised from peripheral, intermediate and central achene morphs was 12.0 ± 0.2 mg, 9.5 ± 0.1 mg and 7.1 ± 0.1 mg, respectively. There were significant differences (*P* < 0.05) in mass of each of the three achene morphs and of the seeds excised from them before and after incubation on wet filter paper. Mass of peripheral, intermediate and central achene morphs increased 123.6 %, 108.4 % and 105.3 %, respectively, and seed mass by 60.8 %, 55.5 % and 53.8 %, respectively.

### Part II: Germination and dormancy

#### Germination of fresh achenes and their excised seeds.

##### Germination of fresh achenes.

**Figure 2.**
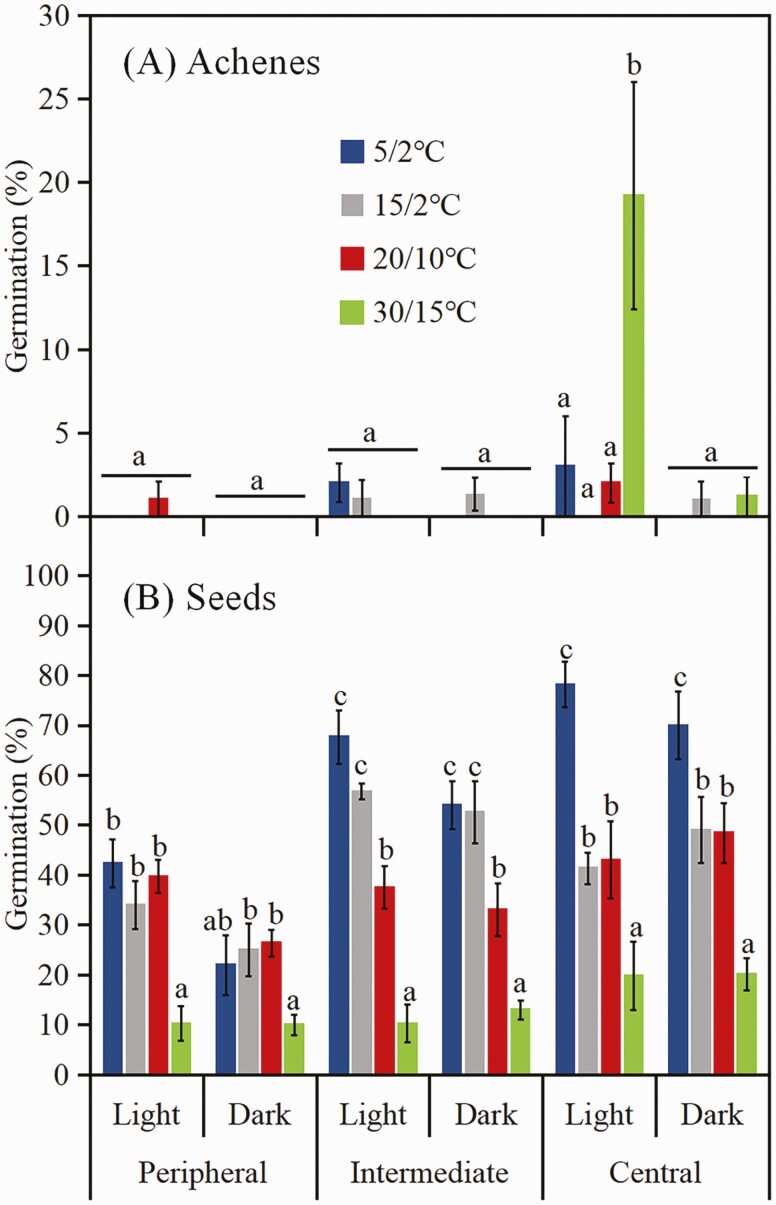
Germination percentages (mean ± 1 SE) of fresh (0 months old) achenes (A) and seeds (pericarp removed from achene) (B) of the three morphs of *Heteracia szovitsii* incubated in light and dark at four temperature regimes. Different lowercase letters indicate significant differences (*P* < 0.05) among different temperature regimes in light/dark or in dark within the same achene morphs.

A three-way ANOVA showed that germination percentage was significantly affected by achene morph, light, temperature and their interactions **[see****Supporting Information—Table S1****]**. The highest germination percentage (19.2 %) was for central achene morphs in light at 30/15 °C (Fig. 2A). However, intermediate and peripheral achene morphs germinated to ≤2.0 % and ≤1.0 %, respectively, regardless of test conditions.

##### Germination of seeds excised from fresh achenes.

A three-way ANOVA showed that germination percentage of seeds removed from the achenes was significantly affected by achene morph, light, temperature and the interaction between achene morph and temperature **[see****Supporting Information—Table S1****]**. Germination percentages of seeds from all three morphs were higher at low than at high temperature regimes in both light and dark (Fig. 2B). The highest germination of seeds from peripheral, intermediate and central achene morphs was 42.4 %, 67.6 % and 78.2 %, respectively, in light at 5/2 °C.

##### Effect of light/dark and temperature on dormancy break of achenes.

**Figure 3.**
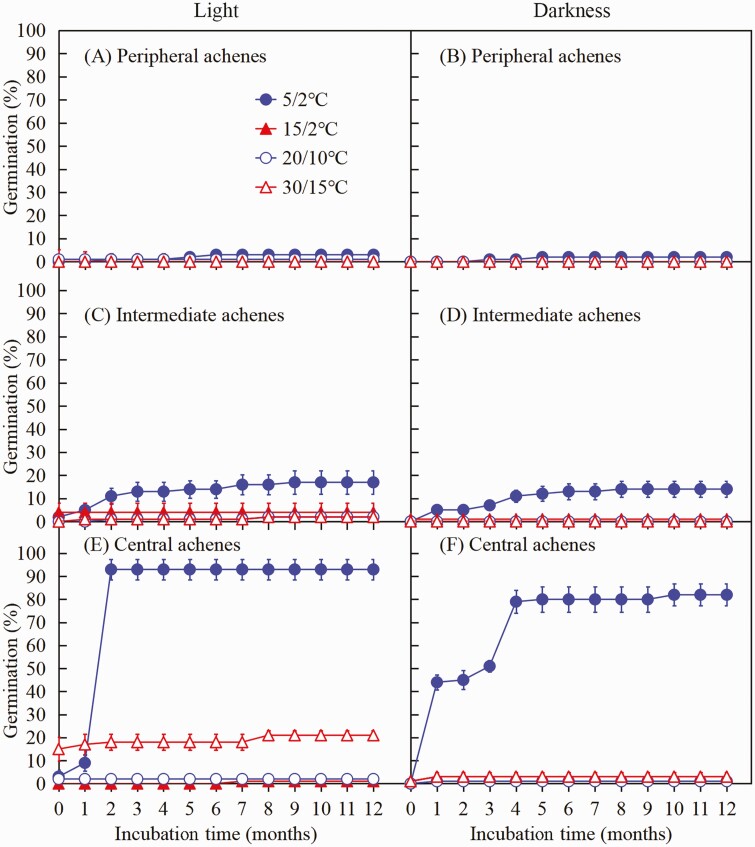
Germination percentages (mean ± 1 SE) of the three fresh achene morphs of *Heteracia szovitsii* incubated at four temperature regimes in light (A, C, E) and in darkness (B, D, F) for 12 months.

A four-way ANOVA revealed significant effects of achene morph, temperature, light, incubation time and their interactions on germination **[see****Supporting Information—Table S2****]**. At the optimum temperature regime (5/2 °C), 3.0 %, 17.0 % and 93.0 % of peripheral, intermediate and central achene morphs germinated in light, respectively, and 2.0 %, 14.0 % and 82.0 % in darkness, respectively (Fig. 3). Germination percentage in darkness was not significant for intermediate (*P* > 0.05) or peripheral (*P* > 0.05) achene morphs at any of the four temperature regimes. However, the decrease in germination percentages in darkness was significant for central achene morphs at 5/2 °C and 30/15 °C (*P* < 0.05).

##### Effect of dry storage (after-ripening) on dormancy break of achenes.

**Figure 4.**
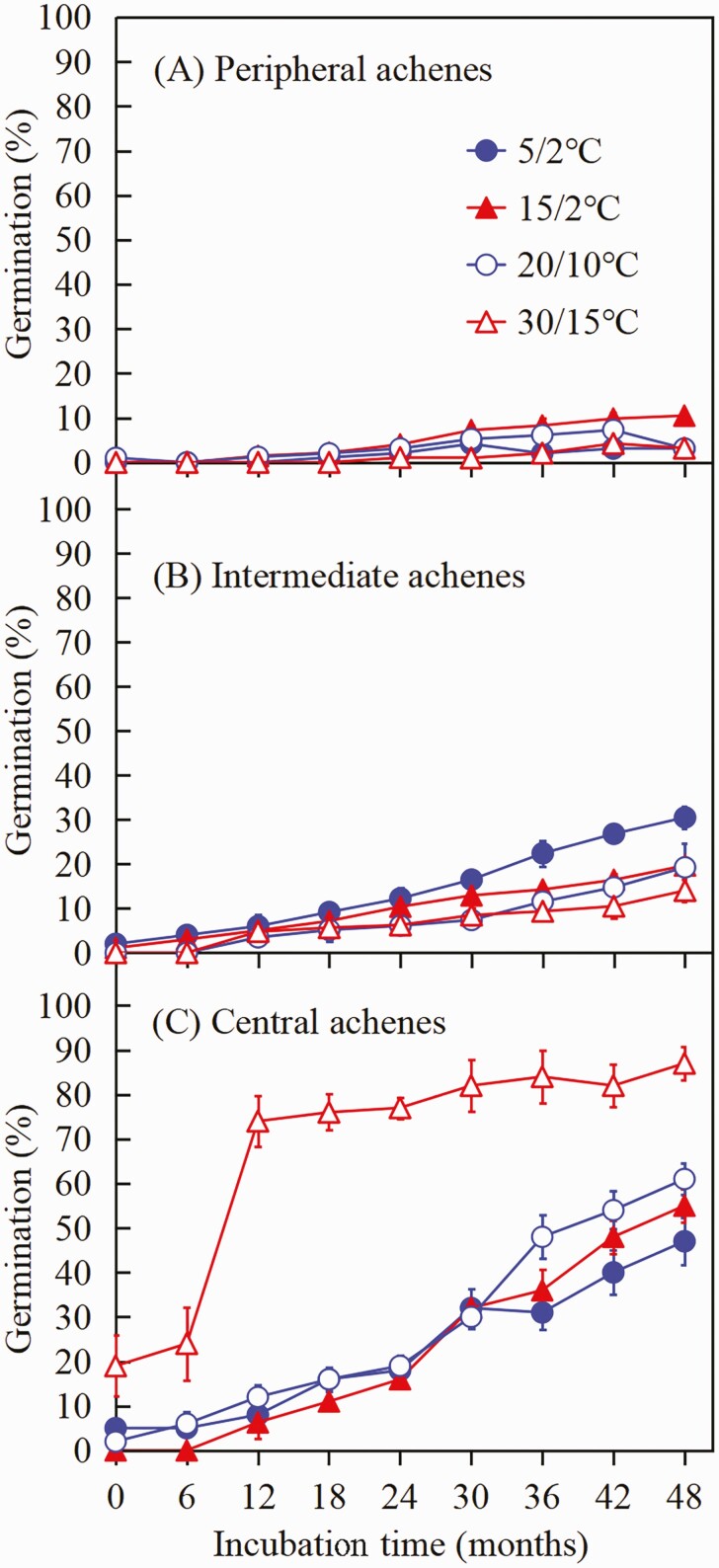
Final germination percentages (mean ± 1 SE) of the three achene morphs of *Heteracia szovitsii* incubated in light at four temperature regimes following 0, 6, 12, 18, 24, 30, 36, 42 and 48 months of dry storage under laboratory conditions.

A three-way ANOVA showed significant effects of achene morph, storage time, temperature and all their interactions on germination **[see****Supporting Information—Table S3****]**. Most of the peripheral (Fig. 4A) and intermediate (Fig. 4B) achene morphs remained dormant after 48 months, and regardless of temperature maximum germination was only 10.5 % and 30.5 %, respectively. Dormant central achene morphs gradually became non-dormant, and thus germination percentage and temperature range over which seeds germinated increased with dry storage time (Fig. 4C).

##### Effect of cold and/or warm stratification on dormancy break of achenes.

A five-way ANOVA showed non-significant effects of achene morph, temperature, dry storage time, stratification time and stratification treatment and their interactions on germination **[see****Supporting Information—Table S4****]**. Thus, neither cold, warm nor warm plus cold stratification was effective in breaking dormancy of achenes of either morph after they had been stored dry at room conditions for 0, 12 and 24 months. The highest germination (2.1 %) was for central achene morphs that received 4 weeks of cold stratification (data not shown).

##### Effect of pericarp scarification on dormancy break of achenes.

**Figure 5.**
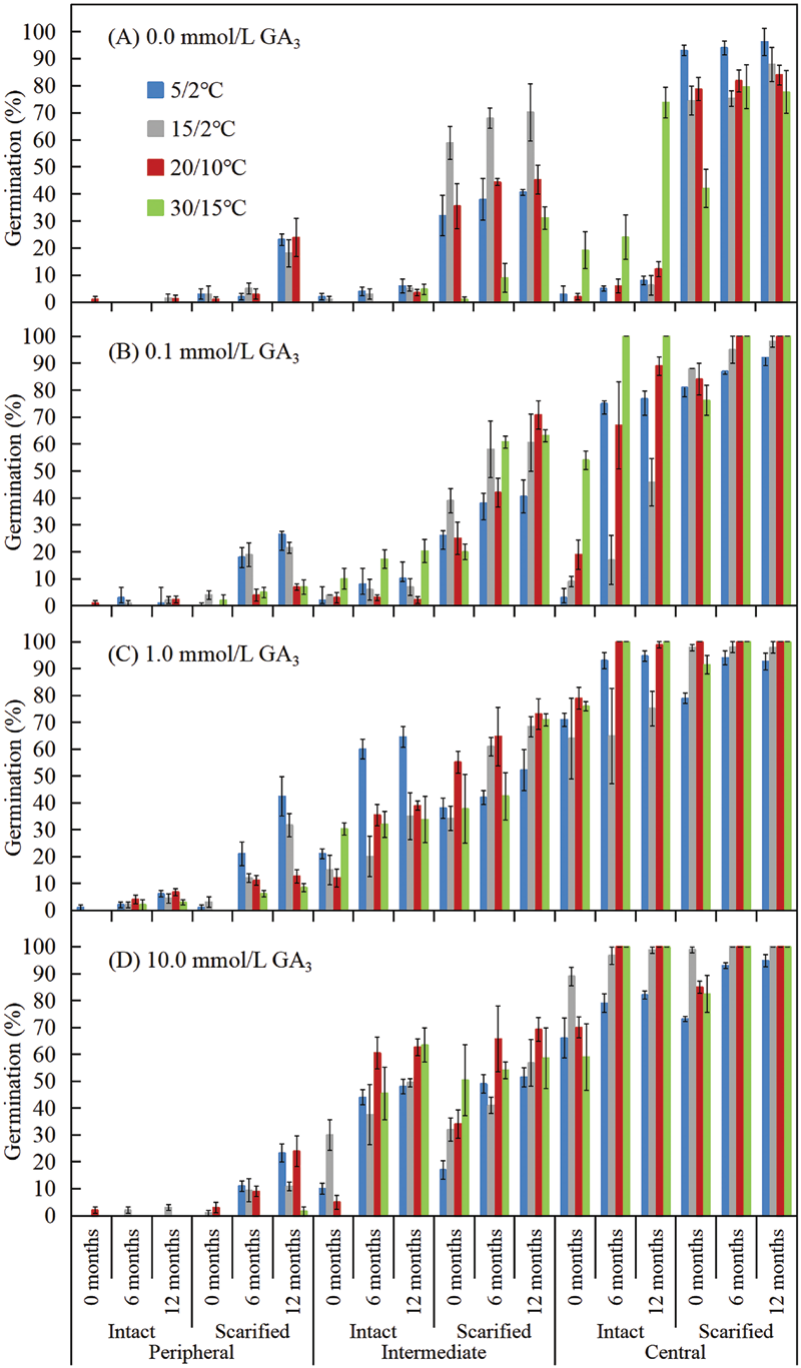
Germination percentages (mean ± 1 SE) of 0-, 6- and 12-month-old intact and scarified (pericarp) of the three achene morphs of *Heteracia szovitsii* during incubation in different gibberellic acid (GA_3_) concentrations at four temperature regimes in light.

A four-way ANOVA showed significant effects of achene morph, temperature, storage time and pericarp treatment (intact vs. scarified) on germination **[see****Supporting Information—Table S5****]**. The highest germination of non-scarified (intact) fresh peripheral, intermediate and central achene morphs was 1.0 %, 2.0 % and 19.2 %, respectively; however, pericarp scarification increased germination to 3.0 %, 58.8 % and 93.0 %, respectively (Fig. 5A). There were significant differences in final germination percentages for intact vs. scarified fresh intermediate and central achene morphs (both *P* < 0.05) but not for peripheral achene morphs (all *P* > 0.05). With increase in time of dry storage, the effectiveness of pericarp scarification in promoting germination increased for peripheral and intermediate achene morphs but not for central achene morphs (Fig. 5A).

##### Effect of GA_3_ and pericarp scarification on dormancy break of achenes.

A five-way ANOVA showed significant effects of achene morph, temperature, storage time, pericarp treatment (intact and scarified) and GA_3_ concentration on germination **[see****Supporting Information—Table S6****]**. Germination percentages of 0-, 6- and 12-month-old achenes of the three morphs were significantly affected by GA_3_ (*P* < 0.05) and pericarp scarification (*P* < 0.05) (Fig. 5). At all GA_3_ concentrations, germination percentages of non-scarified and scarified central achenes were significantly higher than those of peripheral and intermediate achenes (*P* < 0.05). The highest germination (86.7 %) of intact intermediate achenes was for those treated with 1.0 mmol L^−1^ GA_3_ and incubated at 30/15 °C (Fig. 5C). The most effective treatment for breaking dormancy of peripheral achenes was scarification of the pericarp plus 1.0 mmol L^−1^ GA_3_; 12-month-old peripheral achenes with a scarified pericarp incubated in 1.0 mmol L^−1^ GA_3_ at 5/2 °C germinated to 42.4 % (Fig. 5C).

##### Effect of temperature sequence on dormancy break of achenes.

**Figure 6.**
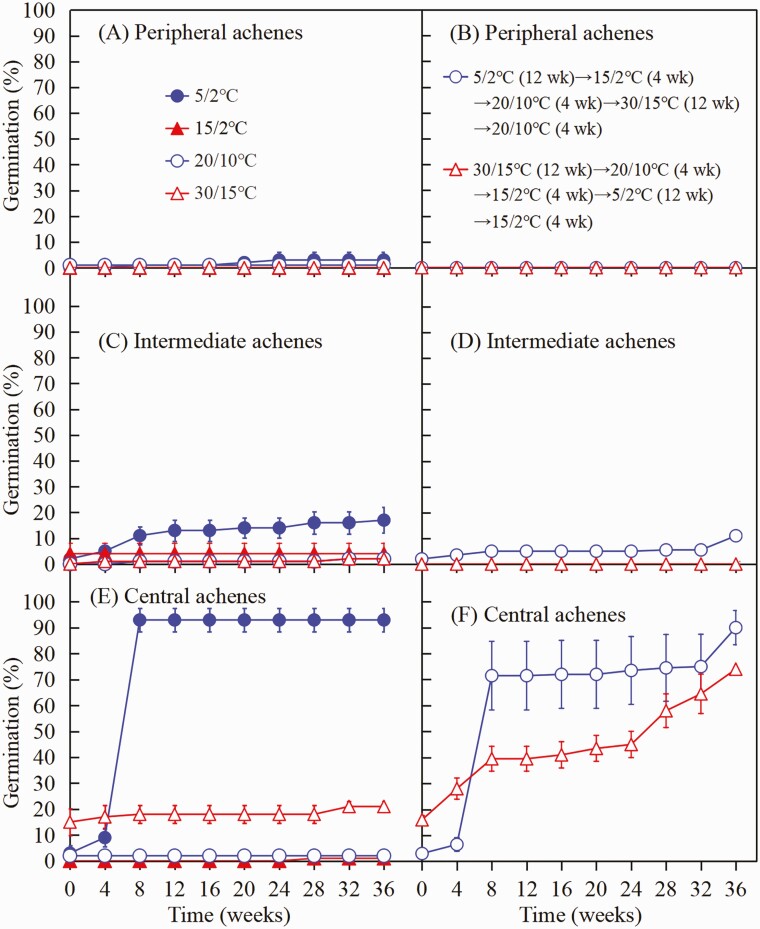
Germination percentages (mean ± 1 SE) of the three achene morphs of *Heteracia szovitsii* in the move-along experiment. (A, C, E) Controls; (B, D, F) move-along sequences (treatments).

In the non-moved controls, peripheral, intermediate and central achenes incubated at 5/2 °C began to germinate after 8, 0 and 0 weeks, respectively, and final germination (36 weeks) was 3.0 % (Fig. 6A), 17.0 % (Fig. 6C) and 93.0 % (Fig. 6E), respectively. However, germination percentages of all three achene morphs were lower at 15/2, 20/10 and 30/15 °C than at 5/2 °C.

In the move-along sequence that began at 5/2 °C, peripheral, intermediate and central achene morphs germinated to 0.0 % (Fig. 6B), 5.0 % (Fig. 6D) and 71.5 % (Fig. 6F), respectively, while they were at 5/2 °C. However, there was no significant increase in germination for either of the three morphs when they were moved sequentially from 5/2 °C → 15/2 °C → 20/10 °C → 30/15 °C. Germination of the intermediate (11.0 %) and central (90.0 %) achene morphs, but not of peripheral achene morphs (0.0 %), increased significantly after they were moved from 30/15 °C to 20/10 °C, the last temperature in the sequence.

In the move-along sequence that began at 30/15 °C, none of the peripheral or intermediate achene morphs had germinated even after 36 weeks (Fig. 6B and D). However, central achenes continued to germinate as they were moved through the whole sequence of temperature regimes, and final germination reached 74.0 % (Fig. 6F).

##### Effect of scarification and dry storage on dormancy break of peripheral and intermediate achenes.

**Figure 7.**
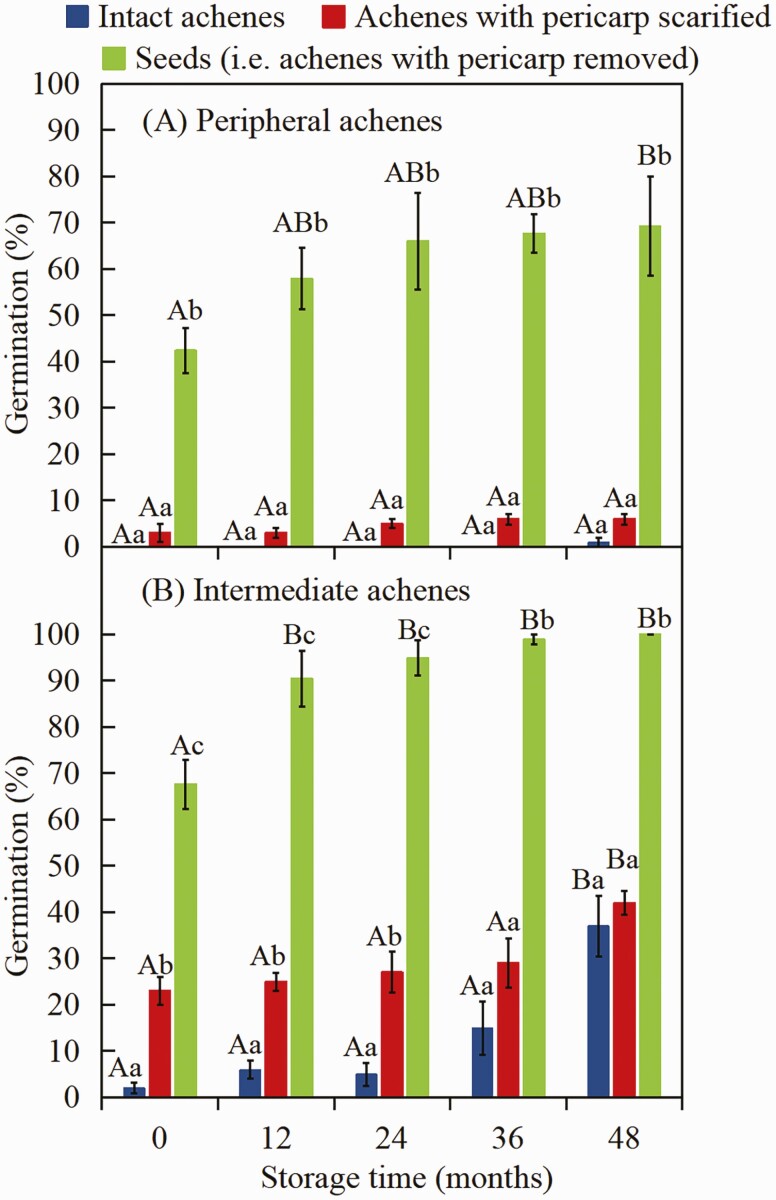
Germination percentages (mean ± 1 SE) at 5/2 °C in light of intact and scarified (pericarp) achenes and of seeds (pericarp removed) of peripheral (A) and intermediate (B) achene morphs of *Heteracia szovitsii* stored (after-ripened) for 0, 12, 24, 36 and 48 months. Different lowercase letters indicate significant differences (*P* < 0.05) among different pericarp treatments within the same dry storage time and different uppercase letters significant differences (*P* < 0.05) among different dry storage times for the same pericarp treatments.

A two-way ANOVA showed that germination percentages of peripheral achene morphs were significantly affected by pericarp treatment **[see****Supporting Information—Table S7****]**. However, storage time and the interaction between pericarp treatment and storage time did not significantly affect germination percentages of peripheral achene morphs. A two-way ANOVA showed that germination percentages of intermediate achene morphs were significantly affected by pericarp treatment, storage time and their interaction **[see****Supporting Information—Table S7****]**. Germination percentages of peripheral (Fig. 7A) and intermediate (Fig. 7B) achene morphs gradually increased with dry storage time in all pericarp treatments. If the pericarp was removed after 48 months of dry storage, peripheral and intermediate achene morphs germinated to a maximum of 69.3 % and 100.0 %, respectively. However, after 48 months of dry storage intact and scarified peripheral achene morphs germinated to only 1.0 % and 6.0 %, respectively (Fig. 7A), whereas those of intermediate achene morphs germinated to 37.0 % and 42.0 % respectively (Fig. 7B).

##### Effect of cold stratification and scarification on dormancy break of peripheral and intermediate achenes.

**Figure 8.**
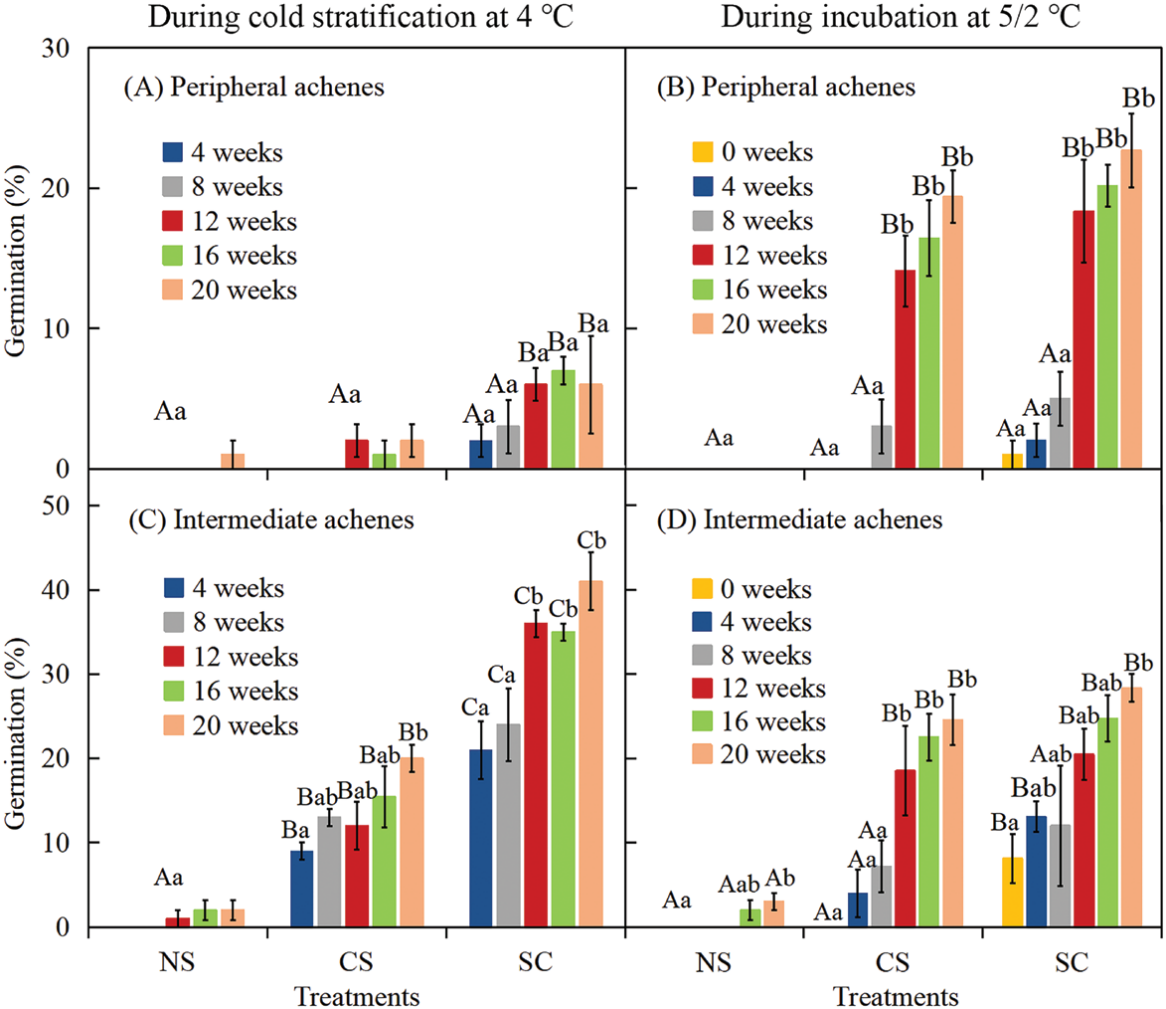
Effect of cold stratification and scarification on germination percentages (mean ± SE) of fresh (0 months old) peripheral (A, B) and intermediate (C, D) achene morphs of *Heteracia szovitsii* during cold stratification at 4 °C for 4, 8, 12, 16 and 20 weeks (A, C) and during incubation at 5/2 °C in light after cold stratification for 0, 4, 8, 12, 16 and 20 weeks (B, D). Different lowercase letters indicate significant differences (*P* < 0.05) among different cold stratification times within the same treatment and different uppercase letters significant differences (*P* < 0.05) among different treatments at the same cold stratification time. NS, intact (non-scarified) cold-stratified achenes; CS, cold-stratified scarified (pericarp) achenes; SC, after intact achenes were cold-stratified, the pericarp was scarified.

A two-way ANOVA showed that germination percentages were significantly affected by cold stratification time, pericarp treatment and their interactions for intermediate and peripheral achene morphs during cold stratification and during incubation at 5/2 °C in light after cold stratification **[see****Supporting Information—Table S8****]**. Compared with intact achenes, however, germination percentages of peripheral and intermediate achene morphs with scarified pericarps increased significantly with increased time of cold stratification (Fig. 8A and C) and during incubation in light at 5/2 °C following cold stratification (Fig. 8B and D). The highest germination for 0-month-old peripheral and intermediate achene morphs with non-scarified and scarified pericarps was 1.0 % and 8.1 %, respectively (Fig. 8B and D). With an increase in cold stratification (achenes that stayed at 4 °C), germination of both morphs with scarified pericarps increased significantly (Fig. 8A and C) and also at 5/2 °C after various periods of cold stratification at 4 °C (Fig. 8B and D). Peripheral achene morphs with pericarp scarified germinated to 6.0 % during 20 weeks at 4 °C (Fig. 8A), which increased to 22.7 % after they were transferred to 5/2 °C after 20 weeks of cold stratification at 4 °C (Fig. 8B). Intermediate achene morphs with pericarp scarified germinated to 41.0 % during 20 weeks at 4 °C (Fig. 8C) and to 28.3 % when transferred to 5/2 °C after 20 weeks at 4 °C (Fig. 8D).

### Part III: Emergence phenology of the three achene morphs in the experimental garden

**Figure 9.**
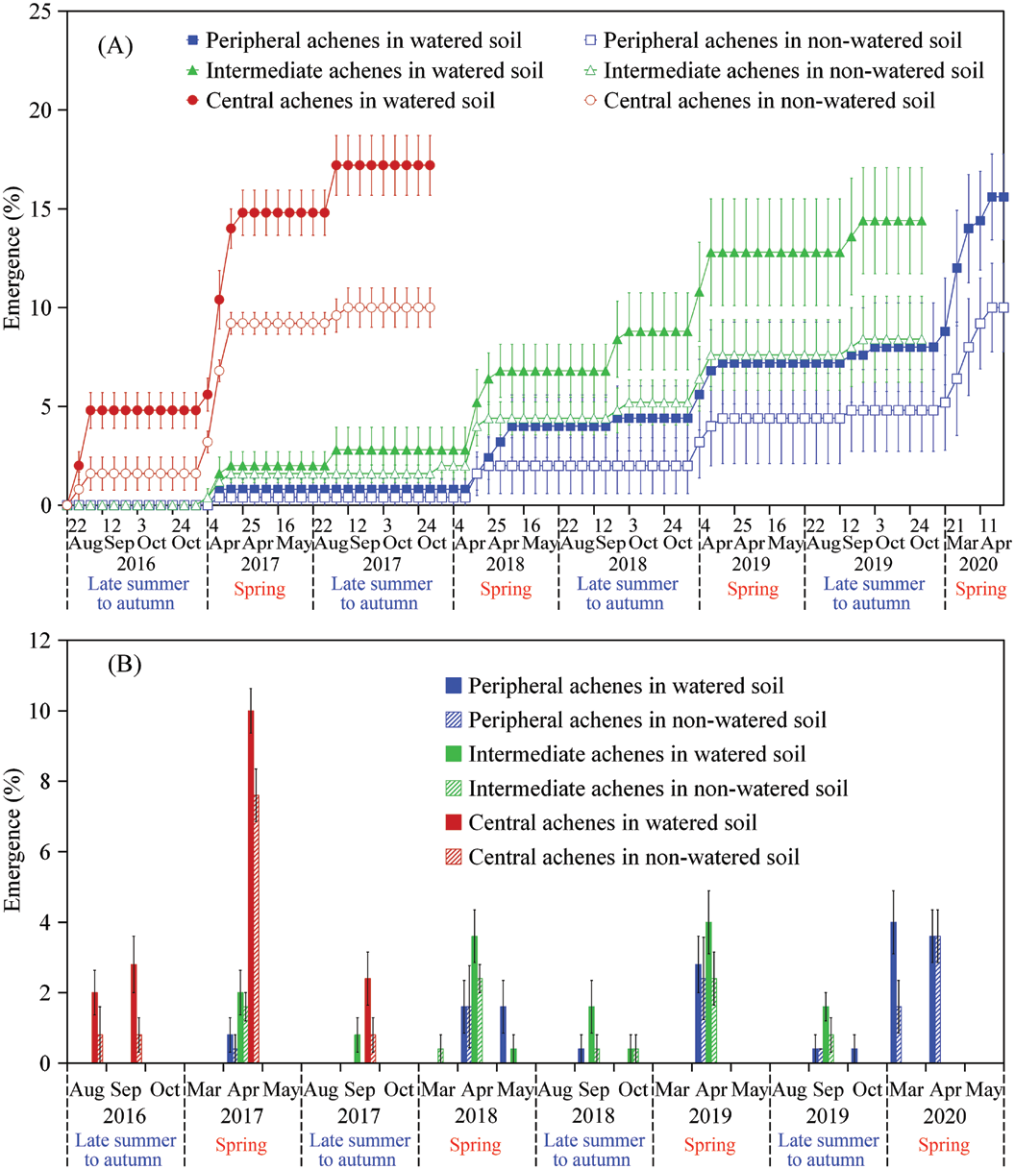
Cumulative emergence percentages (mean ± 1 SE) (A) and emergence percentage (mean ± 1 SE) per month (B) of the three achene morphs of *Heteracia szovitsii* in watered and non-watered (natural precipitation) soil in the experimental garden during autumn and spring 2016–20. Most of the seeds from central and intermediate achene morphs (≥98 %) were non-viable in spring 2018 and spring 2020, respectively.

A three-way ANOVA showed that emergence percentage was significantly affected by achene morph, watering treatment (watered and non-watered) and germination season (autumn and spring) **[see****Supporting Information—Table S9****]**. In autumn 2016, some central achene morphs emerged in watered and in non-watered soil between 29 August and 6 September 2016 (Fig. 9A), when mean daily maximum and minimum temperatures were 26.9 °C and 11.4 °C, respectively **[see****Supporting Information—Fig. S1****]**. Total emergence for central achene morphs was 4.8 % and 1.6 % in watered and non-watered soil, respectively. However, no peripheral or intermediate achene morphs emerged in either watered or non-watered soil in autumn 2016 (Fig. 9A and B).

In spring 2017, most emergence of the three achene morphs in both watered and non-watered treatments occurred from 4 to 25 April (Fig. 9A), when mean daily maximum and minimum temperatures were 17.3 °C and 4.0 °C, respectively **[see****Supporting Information—Fig. S1****]**. For the three achene morphs, emergence percentages in watered soil were higher (but *P* > 0.05) than those in non-watered soil. In both watered and non-watered soil, emergence percentage of central achene morphs was significantly (*P* < 0.05) higher than that of peripheral and intermediate achene morphs, which did not differ significantly (*P* > 0.05). In autumn 2017, emergence percentages of the three achene morphs were similar to that in spring 2017 (i.e. watered > non-watered soil and central > intermediate > peripheral achene morphs in both watered and non-watered soil) (Fig. 9A). Additionally, in both watered and non-watered soil, cumulative emergence percentages of the three achene morphs were significantly lower in autumn than in spring (*P* < 0.05) (Fig. 9B).

In autumn and spring of both 2018 and 2019, some peripheral and intermediate achene morphs emerged, and the results are similar to those in 2017 (i.e. watered > non-watered soil and intermediate > peripheral achene morphs in both watered and non-watered soil) (Fig. 9A). Moreover, cumulative emergence of both peripheral and intermediate achene morphs was significantly higher in spring than in autumn in both watered and non-watered soil (*P* < 0.05) (Fig. 9B). After 47 months, when the experiment ended, only 15.6 % and 10.0 % of the peripheral achene morphs had emerged in watered and non-watered soil, respectively, and only 14.4 % and 8.4 % of the intermediate achene morphs had emerged in watered and non-watered soil, respectively (Fig. 9A).

In both watered and non-watered soil, all non-emerged central and intermediate achene morphs buried to monitor viability were non-viable after 16 (i.e. the spring of 2018) and 40 months (i.e. the autumn of 2019), respectively. However, a high percentage of peripheral achene morphs was still viable after 47 months in watered and non-watered soil, i.e. at the end of experiment in spring 2020.

## Discussion

Depth of dormancy of the three achene morphs was peripheral > intermediate > central, and all three morphs emerged from soil in spring and in autumn. Cumulative emergence percentage of the achenes in the experimental garden was central > intermediate > peripheral. Central achene morphs emerged over a period of ~12 months after sowing, while intermediate and peripheral achene morphs did so for ~40 and 47 months, respectively. No viable central or intermediate achene morphs were present after 16 and 40 months, respectively, but ~60 % of non-emerged peripheral achenes morphs were viable after 47 months. Based on our results, dormancy depth and emergence of the three achene morphs is considered to be a temporal dispersal strategy.

A previous study showed that the dispersal ability of the three achene morphs of *H. szovitsii* was central > intermediate > peripheral (Cheng and Tan 2009). Our study demonstrates that the three achene morphs within a capitulum of this species have PD. However, due to the differences in thickness and anatomical structure of the pericarp (Cheng and Tan 2009) and depth of embryo dormancy (peripheral > intermediate > central), germination in the field and laboratory (central > intermediate > peripheral) differs substantially among the three achene morphs. Thus, the central achene morphs with high dispersal ability and low degree of dormancy have a high risk strategy (H/H), the intermediate achene morphs with moderate dispersal ability and moderate degree of dormancy an intermediate risk strategy (I/I) and peripheral achene morphs with low dispersal ability and high degree of dormancy a low risk strategy (L/L). As such, then, the dispersal/dormancy strategy formula for achenes of *H. szovitsii* is H/H-I/I-L/L (Lu *et al.* 2013; Baskin *et al.* 2014).

In addition to *H. szovitsii*, in the cold desert of northern Xinjiang Province in NW China two other diaspore-trimorphic species, namely *Garhadiolus papposus* (Asteraceae) and *Salsola ferganica* (Amaranthaceae), have the H/H-I/I-L/L strategy. In *G. papposus*, the rank order for wind dispersal ability of central (CA), peripheral (PA) and intermediate (IA) achene morphs was CA > IA > PA, while depth of dormancy was PA > IA > CA (Sun *et al.* 2009; Baskin *et al.* 2014). For *S. ferganica*, diaspores with large (LS), medium (MS) and small (SS) perianth size differed in wind dispersal ability (LS > MS > SS) and degree of dormancy (SS > MS > LS) (Ma *et al.* 2018). Thus, in these two species diaspore dispersal ability/degree of dormancy for a morph also is H/H, I/I or L/L. Three other trimorphic cold desert species have varying dispersal/dormancy strategies: *Atriplex aucheri*, H/H-H/L-L/L (Wei *et al.* 2007; Baskin *et al.* 2014); *Atriplex centralasiatica*, L/H-I/I-H/L (Wang *et al.* 2020); and *Salsola affinis*, H/H-L/H-L/L (Wang and Wei 2007; Baskin *et al.* 2014). Thus, five of the six species with trimorphic diaspores have a high risk (H/H)–low risk (L/L) strategy for diaspore dispersal and dormancy, like most diaspore dimorphic species (Baskin *et al.* 2013).Our first hypothesis that the intermediate and peripheral achene morphs of *H. szovitsii* have intermediate and/or deep PD is supported. As mentioned in the Introduction, PD occurs in three increasing degrees or depths (intensities) of dormancy as follows: non-deep PD < intermediate PD < deep PD. Non-deep PD is broken in seeds of many species by 2–8 weeks of warm stratification (or in seeds of some species by 8–12 weeks of after-ripening in dry storage), but it is broken in seeds/diaspores of other species by 2–10 weeks of cold stratification (Nikolaeva 1969; Baskin and Baskin 2014). Seeds of temperate-/arctic-zone species with intermediate or deep PD require a minimum of 4–24 and 8–25 weeks, respectively, of cold stratification, depending on species, for dormancy to be broken (Baskin and Baskin 2014). However, a pre-treatment period of after-ripening or of warm stratification may reduce the length of the cold stratification period required to break intermediate PD but not deep PD (Nikolaeva 1969). Gibberellic acid usually breaks non-deep PD, may or may not break intermediate PD, depending on the species, and does not break deep PD (Nikolaeva 1969). In fresh (non-treated) seeds with non-deep and intermediate PD, isolated embryos give rise to normal seedlings, while embryos isolated from fresh (non-treated) seeds with deep PD either do not grow, or if they do grow the seedlings are abnormal (Nikolaeva 1969).

The three achene morphs of *H. szovitsii* differed in intensity (degree) of dormancy in the following order: central < intermediate < peripheral. Dormancy break (after-ripening) occurred in most central achene morphs, and they responded well to treatment with GA_3_. Thus, we conclude that the central achene morphs have non-deep PD. However, neither intact intermediate nor peripheral achene morphs responded well to either after-ripening or GA_3_ application. These results suggest that intermediate and peripheral achenes have either intermediate or deep PD. The fact that after-ripened seeds of intermediate and peripheral achene morphs germinated to high percentages after removal of the pericarp and that the seedlings were normal (personal observations by the authors) indicate that neither intermediate nor peripheral achenes have deep PD. Thus, we conclude that the intermediate and peripheral achenes have intermediate PD. However, scarified and intact intermediate achenes responded better to after-ripening and treatment with GA_3_ than peripheral achenes, suggesting that intermediate PD was more intense in peripheral than in intermediate achenes.

Removal of the pericarp significantly increased seed germination of peripheral and intermediate achene morphs of *H. szovitsii* (Figs 5 and 7), suggesting that the embryo lacked enough growth potential (push power) to overcome the mechanical resistance of the pericarp (Baskin and Baskin 2014). Thus, the thick pericarp on the peripheral and intermediate achene morphs may be the reason they are more dormant than the central achene morphs in this species. That is, these structures exert more mechanical restraint on the embryo than the relatively thin pericarp exerts on the embryo of the central achene (Cheng and Tan 2009). This result is consistent with the kind of dormancy reported in Brassicaceae species with indehiscent fruits (Mamut *et al.* 2014; Lu *et al.* 2015, 2017) and peripheral achenes of some Asteraceae species (Tanowitz *et al.* 1987; Sun *et al.* 2009).

Many studies have shown that seeds with non-deep PD exhibit an annual dormancy cycle (i.e. dormant ↔ non-dormant) in which the non-dormant phase of the cycle coincides with the main season of germination (Baskin and Baskin 2014). The three achene morphs of *H. szovitsii* exhibited a peak of emergence in autumn and spring, while no emergence occurred in summer or winter suggesting a 6-month dormancy cycle, like that found in seeds in indehiscent fruits of some Brassicaceae species (Lu *et al.* 2014, 2015, 2017). A 6-month dormancy cycle in *H. szovitsii* ensures that seeds are non-dormant during autumn and early spring, when environmental conditions are likely to be suitable for seedling emergence and plant establishment, and that seeds are dormant in summer and winter, when conditions are unsuitable for germination and seedling growth.

The number of intermediate and peripheral achenes of *H. szovitsii* emerging during each germination season is regulated by (i) the proportion of the seeds of peripheral and intermediate achene morphs in which the embryo has gained enough growth potential to overcome the mechanical resistance of the pericarp and thus can become non-dormant, and (ii) adequate rainfall to moisten the soil continuously for several days. The thick pericarp of the intermediate and peripheral achenes (Cheng and Tan 2009) contributes to the flexibility in germination timing of this species, which may be an adaptation to variation in timing and amount of precipitation in the unpredictable-rainfall habitat in the Junggar Desert.

Our second hypothesis that the three achene morphs produced by a cohort of plants in a given year spread germination over a period of several years, resulting in multiple germination and seedling establishment events, is supported. After dry storage at room temperature for several months, a portion of central achene morphs of *H. szovitsii* had after-ripened. In the field, after-ripening of central achene morphs would occur in summer, and seeds would be non-dormant in autumn, when the soil probably is too dry for them to germinate. Thus, germination of these non-dormant achenes would be delayed until the next spring, when soil moisture (from precipitation plus snowmelt) and temperature are suitable for germination (Wang *et al.* 2006; Liu *et al.* 2014; Bie *et al.* 2016). Indeed, many seedlings were found in spring (late March and April) in the garden. Also we have observed more seedlings in spring than in autumn in the field in several natural habitats of *H. szovitsii*. Since the pericarp delays germination until the second or some subsequent germination season, peripheral and intermediate achene morphs persist in the soil for a longer period of time than central achenes before they germinate. The cumulative germination percentages of intermediate and peripheral achene morphs were very low even 40 and 47 months, respectively, after they were sown in soil (Fig. 9).

Production of different achene morphs within a capitulum likely enables *H. szovitsii* to bet-hedge in the unpredictable-rainfall environment of the Junggar Desert. At maturity, the high proportion of dormant achene morphs of *H. szovitsii* is a mechanism that ensures that all dispersed achenes do not germinate at the same time. In fact, the lack of dispersal and delay of germination of peripheral achenes constitutes a very low risk means of reproduction and thus increases the probability of population persistence of this species. High dispersal ability and reduced dormancy of central achene morphs allow *H. szovitsii* to reach new areas and hence to expand its distribution in space.

## Supporting Information

The following additional information is available in the online version of this article—

Figure S1. Monthly total precipitation and mean minimum and mean maximum monthly temperatures from 2016 to 2019 in Urümqi, China. The weather data were provided by the National Meteorological Information Center, China Meteorological Administration.

Table S1. Three-way ANOVA of effects of achene morph (M), temperature (T), light (L) and their interactions on germination of fresh (0 months old) achenes and seeds of *Heteracia szovitsii* at four temperature regimes in light and dark.

Table S2. Four-way ANOVA of effects of achene morph (M), temperature (T), light (L), incubation time (I) and their interactions on germination of fresh (0 months old) achenes of *Heteracia szovitsii* incubated continuously at four temperature regimes in light and dark.

Table S3. Three-way ANOVA of effects of achene morph (M), temperature (T), dry storage time (S) and their interactions on germination of *Heteracia szovitsii* achenes.

Table S4. Five-way ANOVA of effects of achene morph (M), temperature (T), dry storage time (S), stratification time (S′), stratification treatment (ST) and their interactions on germination of *Heteracia szovitsii* achenes at four temperature regimes in light.

Table S5. Four-way ANOVA of effects of achene morph (M), temperature (T), dry storage time (S), pericarp treatment (P) and their interactions on germination of *Heteracia szovitsii* achenes at four temperature regimes in light.

Table S6. Five-way ANOVA of effects of achene morph (M), temperature (T), dry storage time (S), pericarp treatment (P), GA_3_ concentration (G) and their interactions on germination of *Heteracia szovitsii* achenes at four temperature regimes in light.

Table S7. Two-way ANOVA of effects of dry storage time (S), pericarp treatment (P) and their interactions on germination of peripheral and intermediate achenes of *Heteracia szovitsii*.

Table S8. Two-way ANOVA of effects of cold stratification time (C), pericarp treatment (P) and their interactions on germination of peripheral and intermediate achenes of *Heteracia szovitsii* during cold stratification at 4 °C and during incubation at 5/2 °C in light after cold stratification.

Table S9. Three-way ANOVA of effects of achene morph (M), watering treatment (W), germination season (S) and their interactions on germination of *Heteracia szovitsii* achenes in the experimental garden.

plaa056_suppl_Supplementary_Materials

## Notes

**Populations & Communities. Chief Editor**: Jean Burns
