# Supplementary material for: Dormancy and germination of the trimorphic achenes of a cold desert annual: spreading the risk over time
Source: AoB Plants. 2020 Oct 23;12(6):plaa056. doi: 10.1093/aobpla/plaa056 (PMC7717478; doi:10.1093/aobpla/plaa056)
Supplement: plaa056_suppl_Supplementary_Materials [file plaa056_suppl_supplementary_materials.pdf]

Dormancy and germination of the trimorphic achenes of a cold desert annual: spreading the risk over time

Juanjuan Lu[1], Wenjing Dong, Dunyan Tan[1,*], Carol C. Baskin[1,2,3] and Jerry M. Baskin[1,2]

[1]College of Grassland and Environment Sciences, Xinjiang Agricultural University, Urümqi, China, [2]Department of Biology, University of Kentucky, Lexington, KY, USA, [3]Department of Plant and Soil Sciences, University of Kentucky, Lexington, KY, USA

*Corresponding author's email address: tandunyan@163.com

Short-title: Dormancy in trimorphic achenes

Supplementary Figure S1

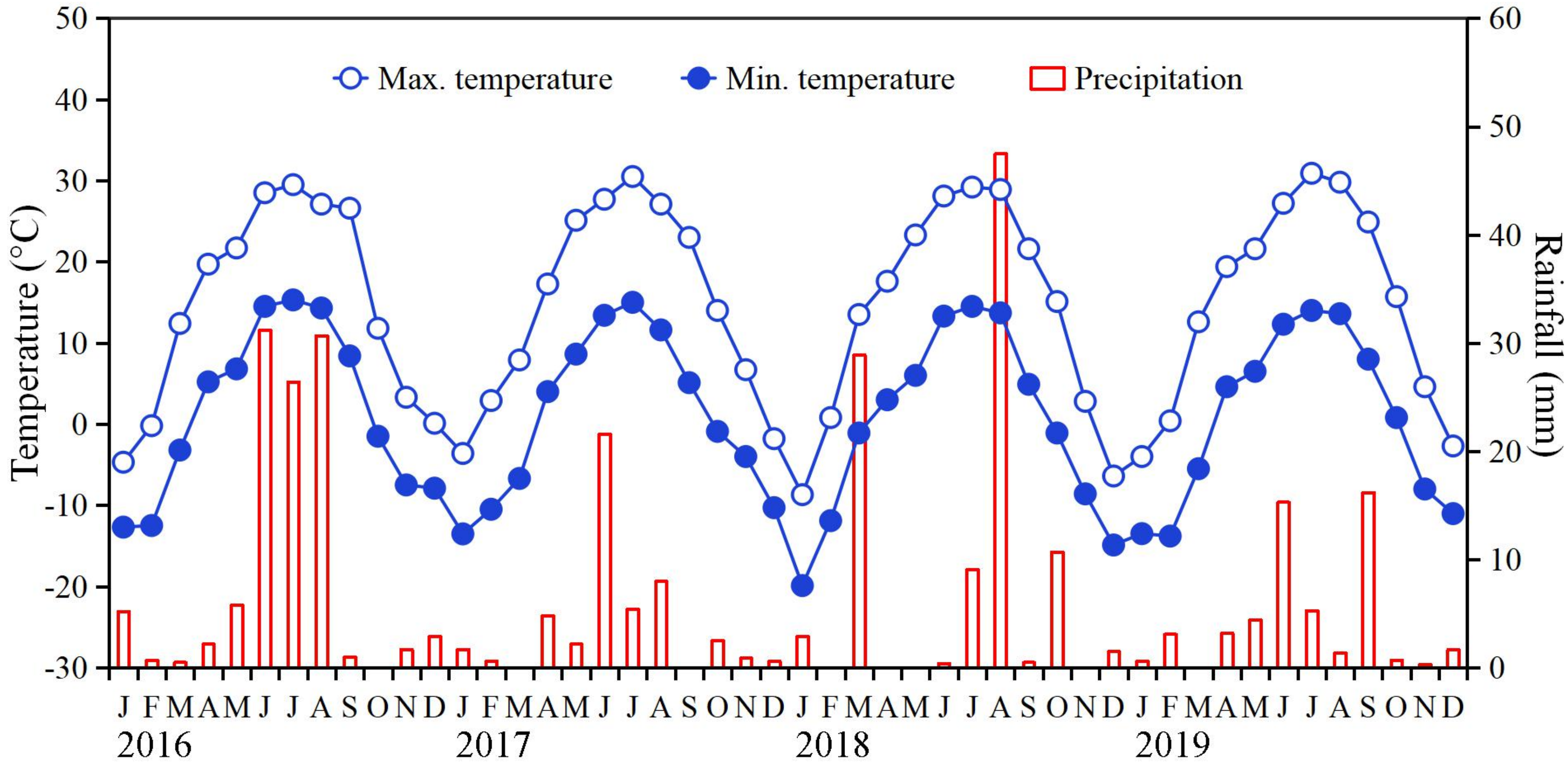

Table S1: Three-way ANOVA of effects of achene morph (M), temperature (T), light (L) and their interactions on germination of fresh (0 months old) achenes and seeds of *Heteracia szovitsii* at four temperature regimes in light and dark.

Factor	d.f.	F-value	P-value
Achenes			
M	2	9.02	<0.05
T	3	4.39	<0.05
L	1	9.85	<0.05
M × T	6	5.93	<0.05
M × L	2	6.53	<0.05
T × L	3	4.21	<0.05
M × T × L	6	4.82	<0.05
Seeds			
M	2	35.64	<0.05
T	3	76.86	<0.05
L	1	5.71	<0.05
M × T	6	7.46	<0.05
M × L	2	3.04	0.054
T × L	3	2.68	0.053
M × T × L	6	0.39	0.882

Table S2: Four-way ANOVA of effects of achene morph (M), temperature (T), light (L), incubating time (I) and their interactions on germination of fresh (0 months old) achenes of *Heteracia szovitsii* incubated continuously at four temperature regimes in light and dark.

Factor	d.f.	F-value	P-value
M	2	3005.65	<0.05
T	3	3145.39	<0.05
L	1	168.56	<0.05
I	12	43.93	<0.05
M × T	6	1941.58	<0.05
M × L	2	66.88	<0.05
M × I	24	25.45	<0.05
T × L	3	37.43	<0.05
T × I	36	37.97	<0.05
L × I	12	4.25	<0.05
M × T × L	6	40.25	<0.05
M × T × I	72	22.43	<0.05
M × L × I	24	3.18	<0.05
T × L × I	36	4.07	<0.05
M × T × L × I	72	3.23	<0.05

Table S3: Three-way ANOVA of effects of achene morph (M), temperature (T), dry storage time (S) and their interactions on germination of *Heteracia szovitsii* achenes.

Factor	d.f.	F-value	P-value
M	2	1566.81	<0.05
T	3	155.19	<0.05
S	8	167.32	<0.05
M × T	6	251.20	<0.05
M × S	16	50.80	<0.05
T × S	24	4.80	<0.05
M × T × S	48	5.14	<0.05

Table S4: Five-way ANOVA of effects of achene morph (M), temperature (T), dry storage time (S), stratification time (S'), stratification treatment (ST) and their interactions on germination of *Heteracia szovitsii* achenes at four temperature regimes in light.

Factor	df	F-value	P-value
M	2	1.24	0.29
T	3	0.73	0.53
S	2	0.73	0.48
S'	5	1.12	0.35
ST	2	0.73	0.48
M×T	6	1.24	0.28
M×S	4	1.24	0.29
M×S'	8	1.28	0.25
M×ST	4	1.24	0.29
T×S	6	0.73	0.63
T×S'	15	1.12	0.33
T×ST	6	0.73	0.63
S×S'	10	1.12	0.34
S×ST	4	0.73	0.57
S'×ST	10	1.12	0.34
M×T×S	12	1.24	0.25
M×T×S'	24	1.28	0.16
M×T×ST	12	1.24	0.25
M×S×S'	16	1.28	0.20
M×S×ST	8	1.24	0.27
M×S'×ST	16	1.28	0.20
T×S×S'	30	1.12	0.30
T×S×ST	12	0.73	0.72
T×S'×ST	30	1.12	0.30
S×S'×ST	20	1.12	0.32
M×T×S×S'	48	1.28	0.09
M×T×S×ST	24	1.24	0.20
M×T×S'×ST	48	1.28	0.09
M×S×S'×ST	32	1.28	0.13
T×S×S'×ST	60	1.12	0.25
M×T×S×S'×ST	96	1.28	0.04

Table S5: Four-way ANOVA of effects of achene morph (M), temperature (T), dry storage time (S), pericarp treatment (P) and their interactions on germination of *Heteracia szovitsii* achenes at four temperature regimes in light.

Factor	df	F-value	P-value
M	2	751.78	<0.05
T	3	8.91	<0.05
S	2	51.73	<0.05
P	1	1619.31	<0.05
M×T	6	26.19	<0.05
M×S	4	3.59	<0.05
M×P	2	363.69	<0.05
T×S	6	4.17	<0.05
T×P	3	77.69	<0.05
S×P	2	5.09	<0.05
M×T×S	12	5.28	<0.05
M×T×P	6	20.31	<0.05
M×S×P	4	6.23	<0.05
T×S×P	6	1.95	0.074
M×T×S×P	12	3.09	<0.05

Table S6: Five-way ANOVA of effects of achene morph (M), temperature (T), dry storage time (S), pericarp treatment (P), GA_3 concentration (G) and their interactions on germination of *Heteracia szovitsii* achenes at four temperature regimes in light.

Factor	df	F-value	P-value
M	2	6406.17	<0.05
T	3	4.98	<0.05
S	2	422.96	<0.05
P	1	1756.76	<0.05
G	3	475.93	<0.05
M×T	6	23.11	<0.05
M×S	4	31.37	<0.05
M×P	2	179.75	<0.05
M×G	6	131.39	<0.05
T×S	6	6.37	<0.05
T×P	3	71.43	<0.05
T×G	9	12.71	<0.05
S×P	2	0.46	0.63
S×G	6	12.11	<0.05
P×G	3	173.65	<0.05
M×T×S	12	7.11	<0.05
M×T×P	6	19.21	<0.05
M×T×G	18	12.18	<0.05
M×S×P	4	28.56	<0.05
M×S×G	12	11.71	<0.05
M×P×G	6	69.82	<0.05
T×S×P	6	4.13	<0.05
T×S×G	18	2.82	<0.05
T×P×G	9	17.19	<0.05
S×P×G	6	2.81	<0.05
M×T×S×P	12	1.71	0.06
M×T×S×G	36	2.50	<0.05
M×T×P×G	18	9.07	<0.05
M×S×P×G	12	10.09	<0.05
T×S×P×G	18	2.55	<0.05
M×T×S×P×G	36	2.71	<0.05

Table S7: Two-way ANOVA of effects of dry storage time (S), pericarp treatment (P) and their interactions on germination of peripheral and intermediate achenes of *Heteracia szovitsii.*

Factor	d.f.	F-value	P-value
Peripheral achenes			
S	4	2.53	0.054
P	2	269.76	<0.05
S × P	8	1.72	0.12
Intermediate achenes			
S	4	21.34	<0.05
P	2	531.89	<0.05
S × P	8	3.23	<0.05

Table S8: Two-way ANOVA of effects of cold stratification time (C), pericarp treatment (P) and their interactions on germination of peripheral and intermediate achenes of *Heteracia szovitsii* during cold stratification at 4 ºC and during incubation at 5/2 ºC in light after cold stratification.

Factor	d.f.	F-value	P-value
Peripheral achenes			
During cold stratification			
C	4	2.19	0.086
P	2	18.88	<0.05
C × P	8	0.75	0.648
During incubation after cold stratification			
C	5	42.26	<0.05
P	2	79.37	<0.05
C × P	10	10.70	<0.05
Intermediate achenes			
During cold stratification			
C	4	11.21	<0.05
P	2	231.78	<0.05
C × P	8	3.38	<0.05
During incubation after cold stratification			
C	5	15.32	<0.05
P	2	55.93	<0.05
C × P	10	2.93	<0.05

Table S9: Three-way ANOVA of effects of achene morph (M), watering treatment (W), germination season (S) and their interactions on germination of *Heteracia szovitsii* achenes in the experimental garden.

Factor	d.f.	F-value	P-value
M	2	13.50	<0.05
W	1	6.56	<0.05
S	1	20.27	<0.05
M × W	2	0.54	0.58
M × S	2	12.36	<0.05
W × S	1	0.00	0.97
M × W × S	2	0.07	0.93
